# Study of the Vertical Structure of the Coastal Boundary Layer Integrating Surface Measurements and Ground-Based Remote Sensing

**DOI:** 10.3390/s20226516

**Published:** 2020-11-14

**Authors:** Teresa Lo Feudo, Claudia Roberta Calidonna, Elenio Avolio, Anna Maria Sempreviva

**Affiliations:** 1 ISAC-CNR Institute of Climate and Atmospheric Sciences—National Research Council, Industrial Area Comp. 15, 88046 Lamezia Terme (CZ), Italy; cr.calidonna@isac.cnr.it (C.R.C.); e.avolio@isac.cnr.it (E.A.); 2 Department of Wind Energy, Technical University of Denmark, Risoe Campus, DTU, Frederiksborgvej 399, 4000 Roskilde, Denmark; anse@dtu.dk

**Keywords:** remote sensing, WRF model, ABL, IBM method, atmospheric stability

## Abstract

The understanding of the atmospheric processes in coastal areas requires the availability of quality datasets describing the vertical and horizontal spatial structure of the Atmospheric Boundary Layer (ABL) on either side of the coastline. High-resolution Numerical Weather Prediction (NWP) models can provide this information and the main ingredients for good simulations are: an accurate description of the coastline and a correct subgrid process parametrization permitting coastline discontinuities to be caught. To provide an as comprehensive as possible dataset on Mediterranean coastal area, an intensive experimental campaign was realized at a near-shore Italian site, using optical and acoustic ground-based remote sensing and surface instruments, under different weather characteristic and stability conditions; the campaign is also fully simulated by a NWP model. Integrating information from instruments responding to different atmospheric properties allowed for an explanation of the development of various patterns in the vertical structure of the atmosphere. Wind LiDAR measurements provided information of the internal boundary layer from the value of maximum height reached by the wind profile; a height between 80 and 130 m is often detected as an interface between two different layers. The NWP model was able to simulate the vertical wind profiles and the eight of the ABL.

## 1. Introduction

The coastal discontinuity, in terms of the thermal and mechanical characteristics of the surface, results in the adjustment of the air masses adapting to the new surface during both onshore and offshore. The modeling of the adjustment of the flow is a challenging issue because it requires high resolution numerical simulations which, in turn, need complete datasets spanning from the surface to the top of the Atmospheric Boundary Layer (ABL) to validate the results. This is crucial for understanding coastal atmospheric processes. Therefore, beside point measurements at a fixed height above the ground measuring mean and turbulent of atmospheric parameters such as wind speed, U and scalars such as temperature T, humidity Q and turbulent fluxes, there is the need to investigate the development of their vertical structure. Light Detection and Ranging (LiDAR) and Sonic Detection and Ranging (SODAR) technologies have been widely used to study the vertical structure of the atmospheric boundary layer, both inland and offshore [1,2,3,4,5,6,7,8,9,10]: sound waves are scattered by the thermal structure of the atmosphere and light waves are scattered at small particles (Mie scattering) or at air molecules (Rayleigh scattering). Doppler LIDARs (or wind LiDAR) are now increasingly used operationally to estimate mean wind speed [11].

Most studies on the reliability of wind LiDAR have been performed over homogeneous terrain or offshore where the columnar distribution of aerosols is generally homogeneous. Peña et al. [12], present results from 10 years of Boundary-Layer research activity at the North Sea coastal site of Hovsore at the west cost of Denmark. During the whole period, several experiments integrated surface and remote-sensing instruments to study the dynamics of coastal flows and LiDAR of different types were used and compared with traditional instrumentation on masts.

Differently from the North Sea, in Mediterranean coastal sites, the local sea/land breeze circulation prevails for most periods of the year [6]. Sea breezes advect over land colder air and marine aerosols with different composition with respect land aerosols; consequently, the vertical distribution of aerosols is often inhomogeneous. Therefore, the integration of both optical and acoustic remote sensing information can help to study these complex interactions.

**Figure 1:**
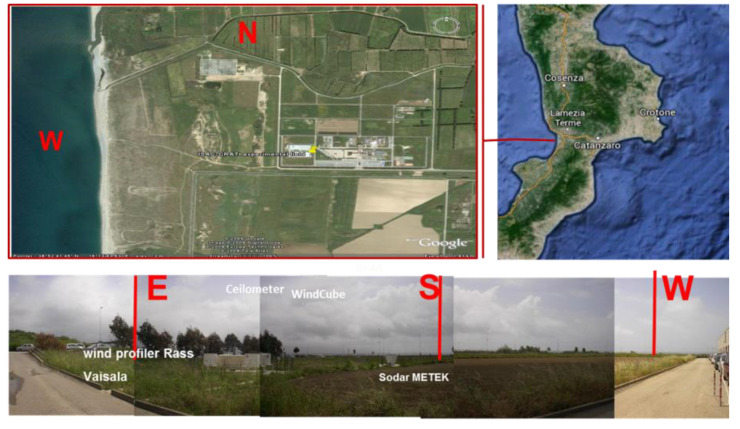
The experimental site and its location, Calabria region in the central Mediterranean area.

Here, we introduce a unique dataset from an intensive experimental campaign at a coastal site in the Central Mediterranean area (Figure 1) during summer 2009. The campaign was organized to investigate the development of the vertical structure of the coastal flow under different meteorological situations; we present a study of the development of the coastal flow, integrating surface mean and turbulent data and datasets from ground-based remote sensing instrument. In particular, surface turbulence measurements provided stability conditions and SODAR and LiDAR provided vertical wind profiles and information about the thermal structure vertical homogeneity of the atmosphere with respect to aerosol content. A CEILOMETER was used to measure the vertical structure of the ABL with respect to the content of aerosols, and high-resolution simulations adopting WRF, a state-of-the-art numerical weather prediction model, was validated.

The paper is organized as follows. In Section 2, we describe the site, the experimental set-up, the methodology and the data set validation used in order to calculate the Height of the Boundary Layer (HBL) using the CEILOMETER backscatter signal and to perform the WRF model-analysis. In Section 3, we present results and discussions. In Section 4, Conclusions, we give final remarks.

## 2. Material and Methods

### 2.1. Site and Instrumental Setup

An intensive experimental campaign was organized in 2009, during the period from July 12th to August 6th, at the coastal infrastructure of CNR-ISAC, section of Lamezia Terme, on the west coast of the Calabrian region (Figure 1) at the southernmost tip of the Italian peninsula in the central Mediterranean (Figure 1).

The CNR-ISAC experimental site (38.88 N, 16.24 E) is located at 600 m from the coastline and 6 m a.s.l, in a flat area in open position into one of three main planes of the region. To estimate vertical profiles of wind and monitor the vertical structure of the ABL, we used different optical and acoustic ground-based remote sensing techniques. Wind LiDAR—WLS7 Windcube—and SODAR—DSDPA.90-24-METEK—allow for the derivation of the vertical profiles of wind speed and direction and of some turbulence characteristics. Furthermore, the vertical profile of the backscatter intensity from aerosol concentration of a LiDAR—CEILOMETER (CL31, Vaisala) allows for the detection of the height of the boundary layer. At the surface, mean and turbulent meteorological parameters were sampled by a METEK Ultrasonic anemometer mounted on a meteorological mast at the height of 10 m.

### 2.2. The Wind LiDAR

The Windcube WLS7 Leosphere is pulsed Doppler LiDAR wind profiler, with a fixed focus, operating at the eye-safe 1.5 µm wavelength. The wind LiDAR sends a train of pulses in five given direction and recording the backscatter in a number of range gates (fixed time delays) triggered by the end of each pulse. The averaged Doppler spectrum obtained for each pulse-stream gives a radial wind speed, i.e., the projection of the wind speed along the line-of-sight.

The measures of radial velocity were performed to four azimuthal directions in the horizontal plane separated by 90° and 15° with the zenith. A full rotation takes about 6 s. The instrument was set to measure at 10 heights from 40 m to 250 m. At each measuring height, the diameter of the rotations was almost comparable to the height. In this frame, especially at the upper level, in presence of advection of air with different aerosol size distribution, the instruments might receive backscatter from air parcels with different speed and aerosol concentration.

### 2.3. The SODAR

The SODAR (DSDPA.90-24-METEK) is an acoustic sounder and provides wind and turbulence vertical profiles in the lower parts of the atmosphere. A back-scatter signal, a small fraction of the acoustic energy from density fluctuations of the backscatter atmosphere, has its frequency shifted according to the wind component parallel to the propagation of the acoustic waves (Doppler effect). It operates ranging from 45 m to 405 m height with a working frequency of 1280 Hz. Sampled data are averaged every 10 min. Acoustic signals are diffused by means of temperature parameter inhomogeneous and the acoustic refractivity index. These signals can be received and the frequency shift can be determined by a sensitive receiver. The distance (or the height range) of the measuring volume can be evaluated by means of the propagation time of the acoustic wave and the estimated acoustic velocity.

### 2.4. The Surface Meteorological Measurements

The 10 m mast with a METEK ultrasonic anemometer, a fast response Hygrometer from NOAA for Turbulent fluxes at 9.5 m, a temperature difference sensors (Risoe-DTU in house) with ΔT_1_ = T5 − T2 and ΔT_2_ = T9.5 − T2, an absolute temperature sensor (Risoe-DTU in house) at 9.5 m and a cup anemometer and wind vane U and Dir at 10 m were used to monitor the surface meteorological and turbulence parameters. The METEK Ultrasonic anemometer were installed and operated routinely within the area of the ISAC research coastal center.

### 2.5. The CEILOMETER

The CEILOMETER CL31 Vaisala was set up for the campaign and located a few meters from the wind cube and from the mast. This instrument transmits laser pulses vertically and measures the backscattered signal that depends on the amount of scattering particles in a volume at a certain distance from the instrument. It operates at a wavelength of 910 nm, has a height resolution of 20 m and a maximum range of 7500 m; it was sounded at zero zenith angles, and data are collected at a frequency of 1 s. Such measures present an uncertainty of ±20% for 30 min averaging periods [13]. In the past, ceilometers have also been successfully employed for detecting the HBL [14,15].

### 2.6. WRF Modeling Approach

The nonhydrostatic WRF-ARW model [16] version 3.4.1 is adopted to simulate the whole measurement campaign. Four two-way nested domains are used. The parent domain covers the whole of Europe (27 km × 27 km); the first nested grid refers to the central-southern Italian Peninsula (9 km × 9 km); the third grid is centered over the Calabria region (3 km × 3 km). Finally, the higher resolution grid (1 km × 1 km) refers to the Lamezia Terme experimental site; this high spatial resolution is adequate to resolve most mesoscale features in the complex study area, and outputs from this domain are taken into account for the comparative analysis. Initial and boundary conditions were obtained from the National Centers for Environmental Prediction—Global Forecast System (NCEP-GFS) analysis (0.5-degree resolution).

The model configuration set-up follows the results of Avolio et al. [17], about the sensitivity of boundary layer variables to different parameterization schemes and Avolio and Federico [18], relative to the simulation of an extreme convective event in the Mediterranean. The main physical parameterizations adopted in this study include the new Rapid Radiative Transfer Model (RRTMG) long wave radiation scheme [19] and the Goddard shortwave radiation scheme [20], the Noah land-surface model [21], the single-moment 5-class microphysics scheme [22] and the Kain–Fritsch cumulus parameterization [23], only for the first two grids. From the above-mentioned previous work [17], it follows that the Yonsei University (YSU) ABL scheme [22] exhibits good performances in simulating the wind vertical profiles and the structure of the ABL; thus, it is the ABL scheme adopted in this work.

To compare WRF model outputs time series and wind LiDAR/SODAR profiles, we extracted the WRF profiles adopting the “nearest neighbor” approach, and interpolated the simulated data at specific vertical levels: 40, 60, 80, 100, 120, 140, 160, 180, 200, 250 m. We chose one-hour instantaneous measurements from the respective data sets. We quantified the performance of the model, compared to the remote sensing sensors, using the standard coefficients and statics methods largely used in literature: Root Mean Square Error (RMSE), Bias (BIAS) and Pearson correlation coefficient.

### 2.7. Methods for Estimating the Height of the Boundary Layer (HBL) from CEILOMETER Measurements

In order to study the evolution of the vertical structure of the boundary layer and its height HBL, we used the CL31 CEILOMETER at a frequency of 10 s. The use CEILOMETER data to estimate the HBL structure were established on several studies; Emeis and Schafer [1] calculated the Height of the Mixed Layer (HML) by identifying the altitude with the minimum backscatter gradient Minimum Gradient Method (GM). Eresmaa et al. [24] and Steyn et al. [25] estimated the HML by fitting an ideal-backscatter profile to the measured backscatter profile; Idealized Backscatter Method (IBM) and GM method divided into different stability classes are reported in [9,14,26].

Then we estimated the height of the boundary layer, using the methodologies, as described in the following session:

#### 2.7.1. The GM and IBM Methods

To estimate the HBL from the backscatter profiles it is necessary to locate the altitude which exhibits the largest negative backscatter gradient. We used the same technique described in [27] and [1].

(1) $$\frac{\partial \beta }{\partial z}\left(z_{i}\right)\approx \frac{\beta \left(z_{i}+\Delta z\right)-\beta \left(z_{i}-\Delta z\right)}{2\Delta z}$$

where *β* is the volume backscatter coefficient, *z_i_* is the height above the ground (a reference height) and Δ*z* = 20 m from the ceilometers setting. The HBL estimates correspond to the minimum of the backscatter gradient calculated using Equation (1). Because fluctuations due to noise in the backscatter profile can result in large noise induced gradients, we smoothed the profiles with a moving averaged filter spanning every 5 measuring-heights before calculating the gradient. These techniques allowed for decreased noise on the gradient calculation, and, to remove it generated by artifacts. The algorithm ignored the first 100 m of the profile.

IBM idealized backscatter profile, using the technique of Steyne et al. [25], was the second method used in this study. The method involves the minimization of the differences between an idealized backscatter profile *B*(*z*) and the observed backscatter profile β(z). The idealized backscatter, *B*(*z*), at height z is given as

(2) $$\;\;B\left(z\right)=\;\;\frac{B_{m}+B_{u}}{2}-\;\frac{B_{m}-B_{u}}{2}erf\left(\frac{z-z_{m}}{s}\right)$$

where *B_m_* and *B_u_* are respectively the mean mixed layer backscatter coefficient and the mean backscatter coefficient immediately above the entrainment layer of depth 2 s, this is a normalization constant related to the entrainment zone thickness (EZT), *z_m_* is the HBL and where the error function erf(x) is defined as $erf\left(x\right)=\;\left(\frac{2}{\sqrt{\pi }}\right)\int_{0}^{x}e^{-t^{2}}dt$.

In the idealized case where the backscatter above the mixing layer shows constant values, B_u_ and B_m_ respectively, the height of the middle of the entrainment layer is defined as the height of atmospheric boundary.

The profile parameters are determined by a nonlinear curve fitting procedure (3), minimizing the least squares β(z) and B(z) and in order to solve the least squares we need to fix the first parameter of *B_m_*, *B_u_*, *z_m_*, *s*, using an initial guess.

(3) $$\;\;Min_{\left(B,\;\;B_{u},\;\;z_{m},\;\;s\right)}\sum i\left(B\;\left(B_{m},\;B_{u},\;z_{m},\;s,\;z\right)-\beta \left(zi\right)\right)$$

#### 2.7.2. The IBM Method and Stability Classes

**Figure 2:**
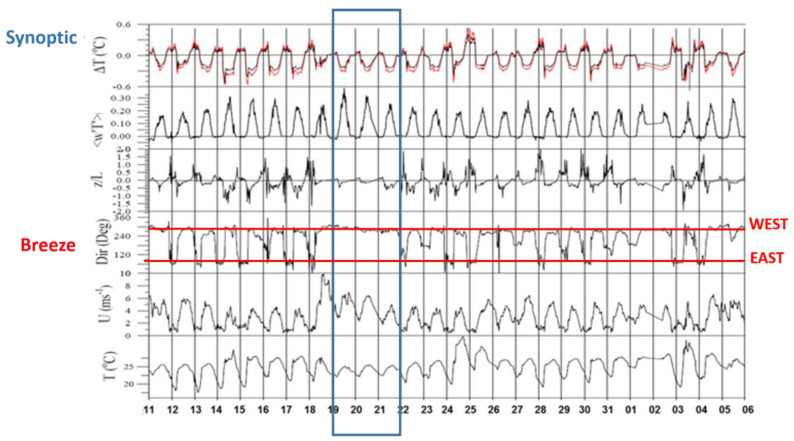
From top to bottom, the time series of, the turbulent heat flux w’T’ (m·s^−1^·K) (note that positive values indicate downward fluxes), the Monin–Obukov z/L, the wind direction (Dir), the wind speed (U), the air temperature (T). The units are indicated in the figure. The area in the blue box corresponds to synoptic flows; red lines delimit the cycle of the complete sea/land breeze in West-East directions.

In this study, we fitted the volume backscatter coefficient, measured with CEILOMETER CL31, with the idealized profile using the IBM method above mentioned, and the idealized profile has the form:

(4) $$\;\;\beta \;=\;\frac{B_{m}+B_{u}}{2}-\;\frac{B_{m}-B_{u}}{2}erf\left(\frac{z-z_{m}}{s}\right)$$

where in this case *B_u_* is the mean backscatter coefficient above the entrainment layer and *B_m_* is the mean backscatter coefficient in the stable, neutral or unstable layer, and s is related to the depth of the entrainment layer. For each weather condition and stability class (given in Table 1 and Figure 2 in the next section and not reported previously) the 10 min backscatter profiles are averaged and a least-squares fit is applied on the average profile using Equation (4). In particular, in this study, we need to set *z_m_* = 400 m, *s* = 100 m, *B_u_* = 4 × 10^−4^ and *B_m_* = 2 × 10^−4^. In our case, we limited measures up 2000 m, although the CEILOMETER can measure up to 7500 m, as a presence of a strong white noise above this quote. For this reason, we considered the first 2000 m the height of backscatter.

During the analysis, rainy hours were removed because the water droplets saturate the CEILOMETER signal and such conditions do not represent cases of convective activity due to the coastal location. For this reason, here we considered only the backscatter profile in clear sky condition and cloud free. We removed all data when in presence of low clouds and when profiles showed an enhanced aerosol backscatter for heights below 1000 m. We carried out a comparison from the two methods and presented the results in the next section.

## 3. Results and Discussion

### 3.1. Weather Conditions during the Intensive Campaign

We observed the evolution of the vertical structure of a coastal flow in different meteorological situations during the campaign. In the area, both synoptic and sea breeze regimes result in westerly winds advecting marine aerosols inland. However, the effect of the advection of the aerosols on the structure of the inland coastal ABL is different in the meteorological situations.

By CEILOMETER it is possible to detect the HBL and we find that during synoptic conditions quote is around 800 m. During sea breeze, HBL shows a daily cycle and its value is typically around 400 m but from backscatter signal it is not possible to distinguish between marine and continental aerosols. Essentially, the high temporal variability of the entrainment process of warm and dry clean air leads to considerable fluctuations of the aerosol concentration and consequently to a large variance in the optical backscatter. Therefore, the height of the maximum variance, and the height of the largest negative peak of the derivative of the optically attenuated backscatter intensity, can both be assumed as HBL [1]. The main characteristics of the atmospheric flow regimes in the area are the following:

- sea breeze and synoptic wind has the same direction i.e., from the West [28];
- during sunny daytime with synoptic forcing, sea breeze is always starting and overimposes on the synoptic winds, conversely, during night the land breeze from the East is suppressed;
- during synoptic regime, the stability conditions are near neutral—due to the higher wind speed than during sea breeze—whereas during the sea land breeze regimes there are the typical unstable-stable daily cycle day and night, respectively.

Later, we will give a general description of the weather conditions during the experimental campaign. This is to provide a preliminary identification of the more evident meteorological situations and to facilitate the identification of suitable case studies that will be described in the following sections. In giving the general weather conditions during the campaign, we considered both the GFS fields used to initialize the WRF model and, to describe the surface conditions, the available surface meteorological measurements. During the campaign, days showed very similar synoptic conditions with the presence of almost exclusively westerly winds. The main observed synoptic structures are a low-pressure area oscillating between the North Atlantic Ocean and the North Sea and a high-pressure area to the North African on the southern Mediterranean. This configuration, almost stationary, has drawn almost exclusively flows from W-NW, both at high vertical levels and at the surface. The main meteorological differences, for the single days, were due to local effects.

The experimental campaign duration, with the complete set of remote sensing instruments, was 23 days, from 15 July to 6 August 2009. From the analysis of the surface meteorological data, shown in Figure 2 (where the days start from 11 July but all the instruments were available from 15 July), we note different type of wind regimes:

1. well-developed sea-land breeze cycle with wind direction shifting between West and East during daytime (sea-breeze with onshore flow) and nighttime (land breeze with offshore flow) respectively;
2. not complete sea-land breeze, i.e., where wind direction during night comes from South, and;
3. synoptic wind flow, from the West (synoptic wind), (Blue box in Figure 2)

From the mast data in Figure 2, we observed a daily cycle in the time series of Temperature differences, turbulent heat flux w′T′ (note that positive values indicate downward fluxes), the Monin–Obukov parameter z/L.

During the night and early morning, the surface layer is stable whilst, during the central hours of the day the surface layer is neutral or unstable, according to the weather conditions. The weather conditions, considering the MAST and the GFS fields (maps not showed for brevity), are summarized in Table 1. Starting from the large-scale analysis (GFS model output), and from the surface conditions (MAST), we performed a first analysis considering all days with a breeze regime and synoptic flow separately.

### 3.2. Comparisons of Observed Wind Profiles from Lidars, Sodars and Simulated Wind Profiles from WRF

The first part of the analysis was performed on the surface-based data set, grouping all days according to well developed sea breeze and synoptic flow, depicted in Figure 2 and Table 1. The days 15, 16, 17, 24, 25, 28, 29, 30 July and 2, 3, 4 August are characterized by a breeze regime well developed with West-East direction, while the selected day’s presented prevalent west wind directions are 19, 20, 21 July. At the same time, for both subdatasets, we analyzed the evolution of wind direction profiles through selected heights of the wind-LiDAR. The results of such analysis confirmed the evolution of the wind flow at all heights (figure not shown). In the second part of the analysis, we compared the vertical wind speed profiles, comparing (i) the wind-LiDAR and SODAR measurements and (ii) wind-LiDAR and the wind speed simulated by the WRF model.

### 3.3. Hourly Wind Evolution during Synoptic Flow and Breeze Regime

**Figure 3:**
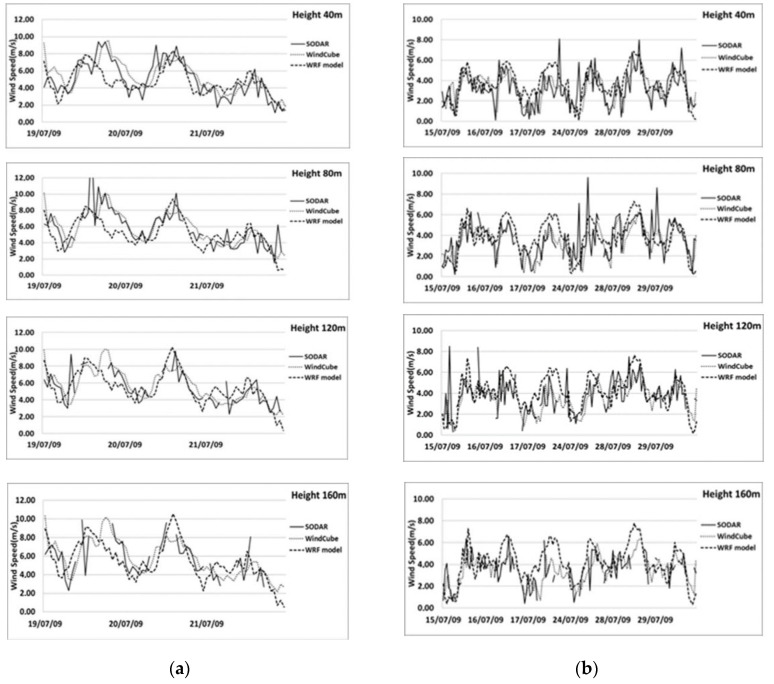
Comparison of observed and simulated daily wind speed profile measured with wind cube, SODAR and WRF model at different heights (40 m, 80 m, 120 m, 160 m) for synoptic flow (**a**) and breeze well developed (**b**). Gaps in solid and dotted lines indicates missing data.

In this study, we analyze the temporal evolution and difference in wind profile among breeze regime and synoptic days. Figure 3 illustrates the comparison of the diurnal evolution of wind speed for different weather conditions, at different heights, using both remote sensing instruments and simulated data.

In the site, both synoptic and sea breeze regimes result in westerly winds; during synoptic flow, the wind predominantly blows from the West (270–300°) (Figure 2) with speeds higher than 4 ms^−1^. Both instruments exhibit a maximum around the central hour of the days (Figure 3a,b). During consecutive days of stationary sea-land breeze regime (Figure 2 and Figure 3b), we observed that after the onset of the sea breeze at around 10:00 local time, the wind speed reached the minimum value obtainable and then intensity increases and becomes stationary around 14:00 local time.

The wind speed simulated by WRF is in general agreement when compared to observations; the daily cycle of the wind speed appears well reproduced, at different heights, although a general underestimation is visible.

In the next section, we discuss the statistics to evaluate the performance of the model.

### 3.4. Comparison between LiDAR and SODAR Wind Profiles

First, we compared LiDAR and SODAR wind speed profiles. Table 2 shows the available SODAR and LiDAR datasets. The data quality check at each height resulted in removing about 2/3 of the data. As the measuring heights of SODAR and LiDAR do not coincide, for each LiDAR measurement height, we calculated the Pearson’s correlation coefficient with both the SODAR time series taken at the levels above and below. Table 2 shows the results.

Table 2 shows that the correlation is best up to 80 m range and that the amount of available data decreases with increasing heights. This is likely due to the different measuring principles of the SODAR and Wind-LiDAR. Above 180 m, the two instruments are only correlated when the wind LiDAR is not disturbed by the advection of marine aerosols.

Considering the several missing data and/or outliers in the SODAR dataset, the comparison with WRF, presented in the next section, has been performed using only the wind LiDAR measurements.

### 3.5. Comparison between LiDAR and WRF Model

In order to evaluate the performance of the WRF model, we compared the wind speed vertical profiles from the LiDAR and the output of the model interpolated at the levels of the wind LiDAR. We used the parameters described in paragraph 2.6, performing a statistical analysis.

**Figure 4:**
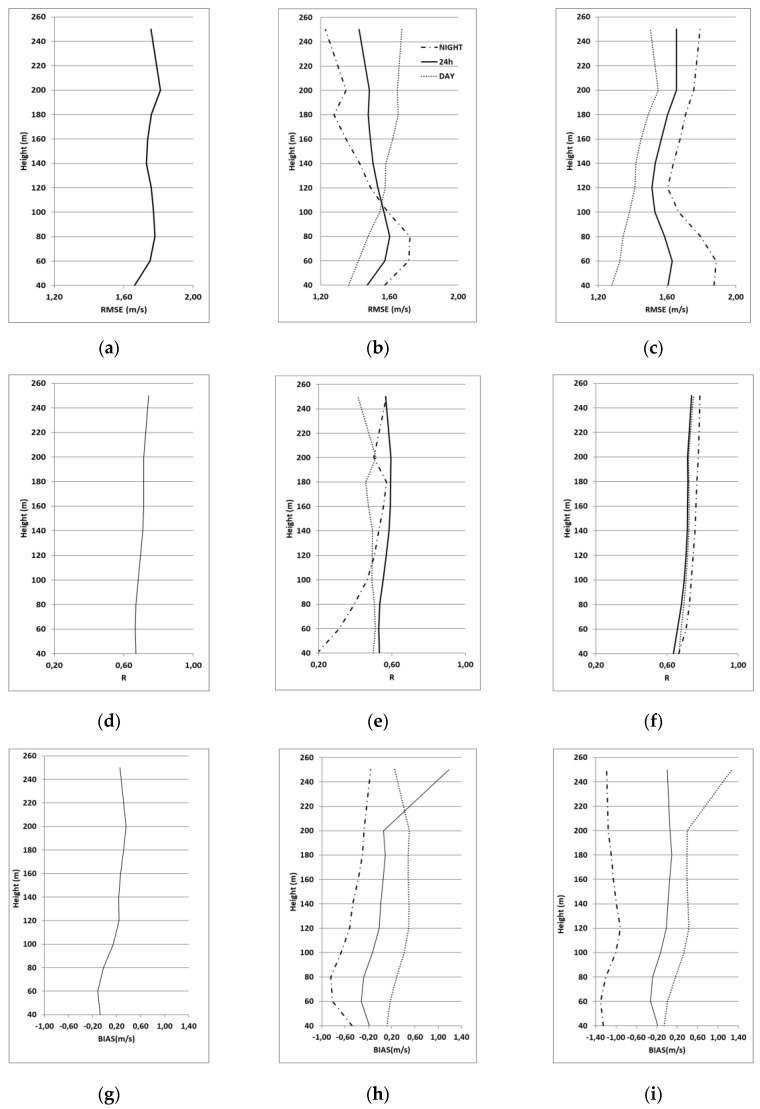
Vertical profiles of RMSE, R and BIAS between wind speed measured by the LiDAR and wind speed simulated by WRF. All days (**a**,**d**,**g**), breeze day (**b**,**e**,**h**) and synoptic flow(**c**,**f**,**i**) daytime and nighttime cases line (all 24 h).

For all days, Figure 4a,d,g, sea breeze Figure 4b,e,h and synoptic regimes Figure 4c,f,i, classified further in daytime and nighttime to evaluate the ability of the model in reproducing the daily cycle behavior.

Figure 4 shows the vertical profiles of the RMSE, R and BIAS respectively.

*Correlation.* The correlation between the model and the LiDAR profiles is moderate-to-high in almost all cases. Values greater than 0.6 are evident for the synoptic cases (without distinction from night and day) (Figure 4f) and for the whole campaign-averaged case (Figure 4d).

In breeze cases (Figure 4e) the correlation is generally lower, with values between 0.5 and 0.6 for the 24 h and during daytime, while very low values of R were obtained during nighttime, below 120 m.

*RSME.* Considering the complete experimental campaign, the vertical profile of RMSE shows values ranging from 1.6 to 1.8 m/s, with two relative maxima at 60–80 m and at 200 m (Figure 4a).

In case of synoptic flow (Figure 4c), the profiles of RMSE are similar in shape to the campaign-averaged case, but values have different behaviors, with better performances during daytime than during nighttime. For the breeze days (Figure 4b), we noted the different evolution of RMSE during diurnal and nocturnal cases. During daytime, the RMSE increases with the heights (similar to the other cases), due to the strong variability of the wind speed when the breeze starts and during its development; during nighttime the decrease of the error with increasing heights is evident, in particular above about 100 m, plausibly associated with the stable layer.

*Bias.* Averaging all days, we found that below 80 m the bias is negative, although small, while in the upper levels, the bias is positive (Figure 4g). Similar behavior is found for the 24 h (the height of 120 m separates negative and positive biases), for the cases of sea breeze (Figure 4h) and synoptic flows (Figure 4i). In both cases, a sensible overprediction (underprediction) of wind speed simulated by WRF is evident at all levels during daytime (nighttime).

### 3.6. Boundary Layer Height Detection by a CEILOMETER and Its Impact on the Wind LiDAR Maximum Profile Heights

Here, we refer to Section 2.7 where we described the methodology used to estimate the HBL during different stability classes and different weather conditions from the CEILOMETER measurements. To reduce the noise in the backscatter signal, we used the Range Corrected Signal (RCS) methodology, developed for aerosol LiDARs [29]; furthermore, we used only cloud free days, in order to not “contaminate” the aerosol profiles by adding multiple peaks and drops to the signal.

Then, with different methodologies, minimum gradient method and idealizing backscatter profile (Section 2.7.1 and Section 2.7.2), we calculated the HBL in different weather conditions (well-developed breezes and synoptic flow).

**Figure 5:**
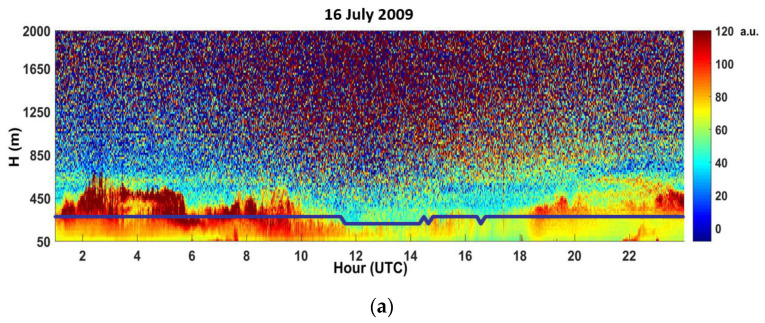

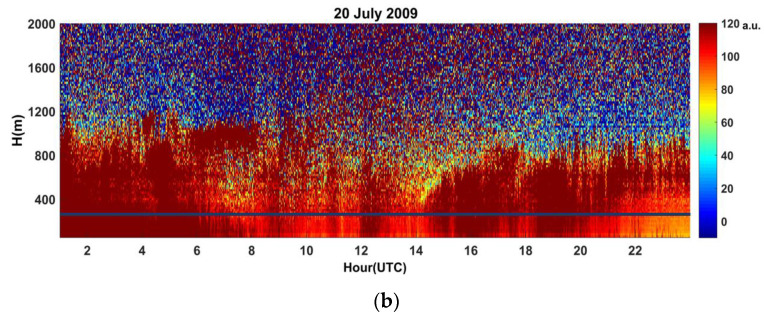
CEILOMETER CL31-10-min average of the intensity of volume backscatter coefficient (in arbitrary units) and also the wind LiDAR maximum obtainable measuring height (blue line) during a breeze day—16 July 2009 (**a**) and synoptic flow day—20 July2009 (**b**). The figure shows that the advection of cleaner air with marine aerosols from the sea with respect to the land aerosols, flowing above the IBL, after the breeze onset, causes a vertical discontinuity of aerosol concentration and thus a reduction of the LiDAR vertical range (**a**).

Figure 5 shows an example of 10-min average profiles of backscatter volume coefficients and the wind LiDAR maximum obtainable measuring heights during one day with breeze (Figure 5a) and one day with synoptic flow (Figure 5b). In Figure 5a, the backscatter shows a nonhomogeneous vertical air column due to the sea-breeze advection of marine aerosols (from 11:00 am to 19:00 pm). While in Figure 5b a more homogeneous layer is present due to higher and constant synoptic flow.

### 3.7. Stability Classes and the Boundary Layer Height from the Ceilometer

We proceeded with analyzing the noise signal and performing the classification of the backscatter signal according to atmospheric stability conditions estimated by the sonic anemometer [5,6,7]. This methodology was applied to the 10-min average of the backscatter profiles in synoptic and breeze condition, according to day and night. Table 3 shows the HBL estimated in both synoptic flow and breeze regime, during night and day, using GM [1,24], IBM [1,9,24] and the IBM method classified in stability classes [24,25]. According to Section 2.7.1 and Section 2.7.2, we used the fix parameters, for the values obtained in Table 3, to be able to apply the two methodologies in different regimes and stability conditions. There is a small difference between the values of HBL estimated from the two methods.

In case of breeze, the resulting values of HBL vary as expected following the daily cycle over land, i.e., low during the stable night and high during the unstable days: during synoptic flow, the HBL is constantly higher than during unstable condition. We note that during the night, synoptic HBL is larger than during the day. A reason can be that during summer the breeze always develops, adding and modulating the synoptic flow (Figure 2, days 18–19–21 July). A well-developed breeze, adding speed, would produce a large quantity of marine aerosols advected on land flowing over the land; aerosol layer would contribute to the detection of a lower HBL.

**Figure 6:**
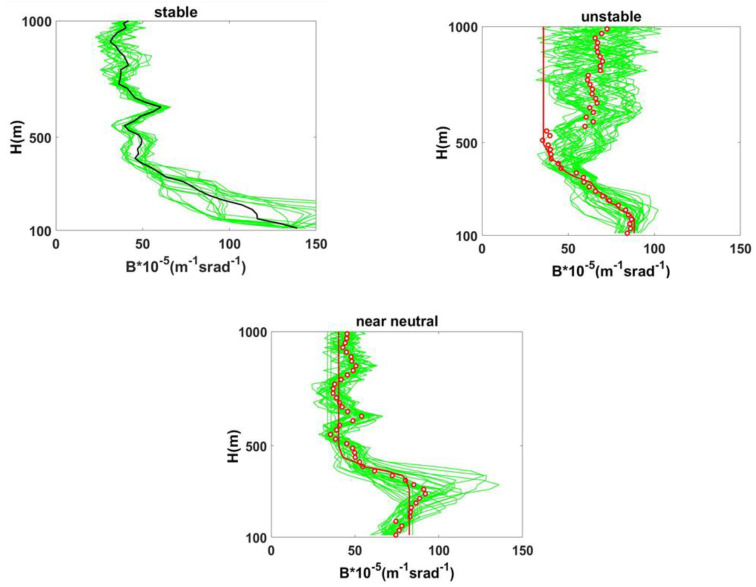
Volume backscatter coefficient, B, as a function of the height, for different stability conditions: Breeze day (**Top**), stability classes: stable (**Top-left**) and unstable (**Top-right**); Synoptic flow near-neutral class (**Bottom**). The backscatter coefficients are shown in green lines, the mean of all signals in red circles and the mean of all signals for “stable” class in black line. The red lines are the fit using IBM profiles [25].

Figure 6 shows an example of the analysis of the CEILOMETER data of volume backscatter coefficients, B, (10 min average), for different stability conditions. In this case, we analyzed the backscatter profiles in stable, unstable and near-neutral stability classes during breeze and synoptic flow days. The daily cycle was not observed during the synoptic flow conditions. In addition, here, during the sea breeze regime, the surface layer shows the classical inland stability daily cycle, unstable during the day and stable during the night. The differences of the height of the HBL internal boundary layer associated on different weather conditions is evident in unstable (up to 170 m) and in stable (below 100 m) condition.

### 3.8. Comparison of the HBL from Observations and from WRF

We compared the HBL resulting from the GM and IBM to the HBL from the WRF model outputs. In this case, we considered the days when the synoptic flow is dominant and the days when the breeze regime is well developed for both at 12 UTC. This hour corresponds, on average, to the maximum value of the HBL height during daytime, very close to the time of maximum insolation. Table 4 shows the results of analysis.

The mean values simulated by WRF are in good agreement with the observed ones for both proposed methods, during breeze. During synoptic conditions, WRF agrees best with the IBM method.

### 3.9. Distribution of Aerosol Concentration and Data Reliability from Wind LiDAR: Carrier to Noise Ratio Analysis

To find the correlation between the maximum height, measured by Wind LiDAR, and the concentration of aerosols along the laser beam path, it is possible to apply another methodology. It consists in using unavailability of measures at a particular height if the aerosol concentration in not homogeneous in the profile. Figure 5, Section 3.6 (blue line) shows an example of the Wind LiDAR maximum obtainable measuring heights in a breeze condition day, due to the presence of the Internal Boundary Layer (IBL) including marine aerosols. In such a case, the aerosol concentration is not homogeneous in the profile and the signal decrease. During days of well-developed sea-land breeze, after the front passage of the sea breeze at around 10:00 UTC, see in Figure 5, the wind LiDAR maximum obtainable measuring height is often limited to 180 m. However, as the sea breeze intensity increases and becomes stationary around 14:00 UTC, the maximum height reached by the Wind lidar increases again (up to 350 m).

These preliminary results suggested that we should focus on the response of the Wind LiDAR in days when the breeze is well developed and at its onset. The main hypothesis is that during these events, the concentration of aerosols in the vertical layer is not homogeneous; therefore, the signal is weak.

We also analyzed the reliability of the wind LiDAR measurements using the CNR of the laser signal. The wind LiDAR disregards all measurements when CNR < −22 dB. This threshold was recommended in [9,26] and used in [30,31].

**Figure 7:**
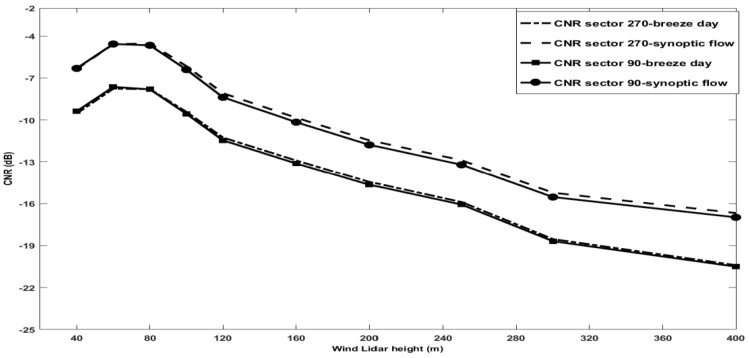
Ensemble average of CNR signals as function of height and for sector 270 ° and 90 ° of the laser beam, during breeze and synoptic flow regime days.

In Figure 7, we illustrate the ensemble average of the CNRs signals as function of height and per sector 270° and 90° of the laser beam, during days with breeze and synoptic flow, and we also noted the differences of about 4dB from two different weather conditions. During a synoptic flow, the CNR curve is higher than during breeze regime; this amount is due to of higher aerosol vertical distributions with the constant blowing wind from the west. Figure 7 shows that the CNR curves for the two azimuthal position, of interest in our case, peak at the focus distances around 80 m and their very close behavior suggests no systematic interference of hand targets or any other source of beam degradation.

**Figure 8:**
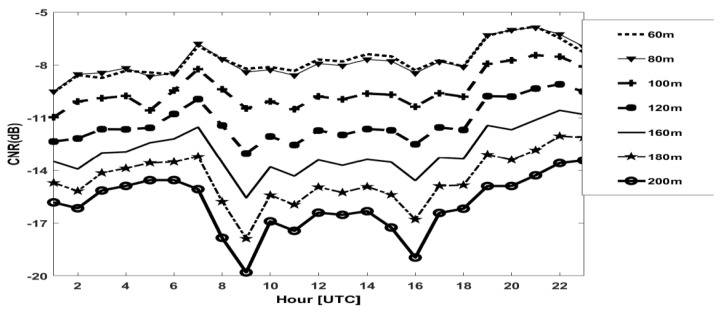
Diurnal cycle, hourly mean of CNR signals at different heights for sector 270 °during breeze regime.

Figure 8 shows the diurnal cycle of the hourly mean of the CNR at different heights averaged over all breeze days. Up to 100 m, the shape of signal is constant at all heights all day long. Above 120 m the signal remains constant and similar at the lower level until the early morning and in the late afternoon. Starting from about 10:00 am, the CNR signal above 120 m remains constant until 16:00 and increases from 16:00 pm to 21:00 pm. CNR at intermediate levels are similar. This might signify the existence of three different layers during the day.

### 3.10. SODAR Analysis: Reflectivity and Standard Deviation of the Vertical Wind Velocity, σ_w_ in Different Meteorological Conditions

Since the working principle of the SODAR is based on density fluctuations due to the thermal structure of the Boundary Layer [32], here, as example, we show the reflectivity according to different meteorological conditions to highlight the thermal effects on surface. Reflectivity surface plots (sodargrams), of the z antenna represent the time series of the profiles measured for the vertical beam direction (zenith = 0°) and a frequency of 1.3 kHz.

**Figure 9:**
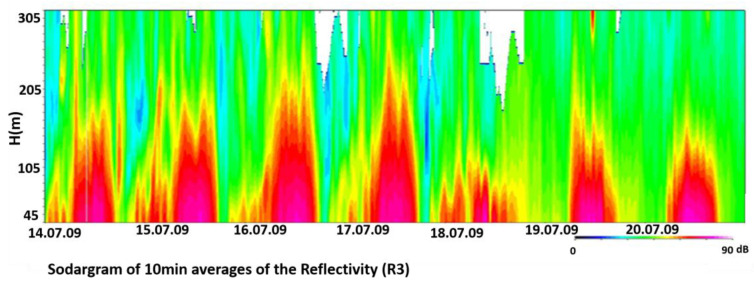
Sodargram of reflectivity from 14/07/2009 to 21/07/2009 during sea breeze (15, 16, 17 July) and synoptic flow (19, 20, 21 July) at Lamezia Terme site.

Figure 9 illustrates, as a sample, the sodargram of the time series of backscatter profiles (10 min averages) in Db of the reflectivity of the radial 3 antenna (vertical) during the period 14 July–20 July 2009 with both sea breeze and synoptic conditions. Measurements that have not passed the error checks successfully are left blank.

During large-scale flow, nighttime stability is toward neutral conditions and therefore the SODAR signal of reflectivity is lower than breeze cases [7,33]. During breeze regime in day-night (unstable-stable) and night-day (stable—unstable) transitions, a strong variability of reflectivity signal is observed during the nighttime and when the breeze starts. This activity in strong stability is likely due to intermittent turbulence.

**Figure 10:**
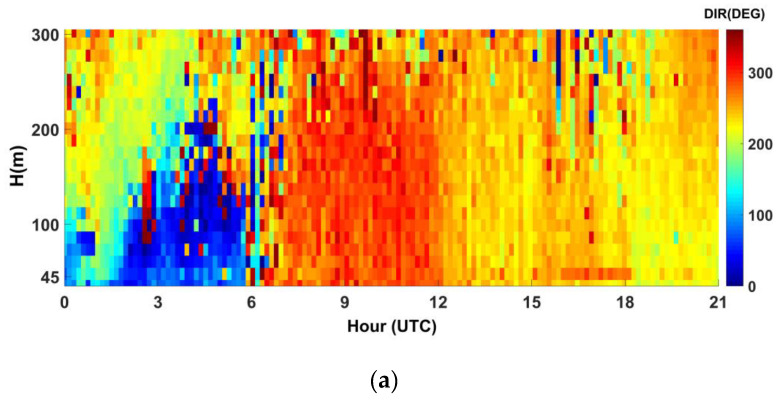

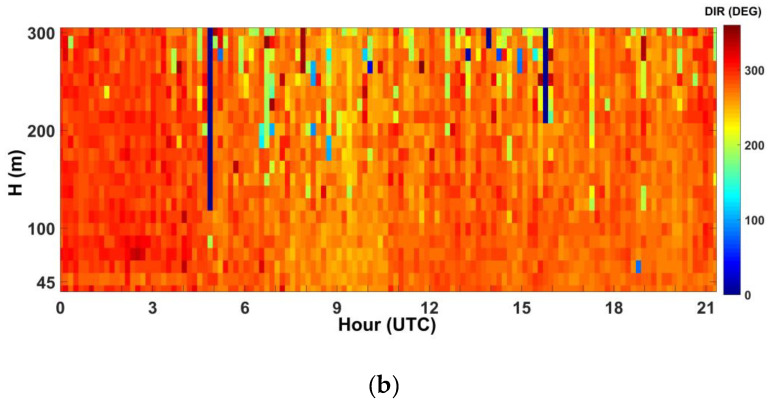
Wind direction measured with SODAR for 15 July 2009 (**a**) and 19 July 2009 (**b**) during sea breeze regime and synoptic flow at Lamezia Terme coastal site.

In Figure 10 we report an example of wind direction measurements with SODAR for 15 July 2009, during sea breeze regime, and 19 July 2009 during synoptic flow; we note the change in direction during the early hours of the morning in breeze cases. The breeze front passage is expressed in wind direction change and increased wind speed after a relatively calm period.

With the help of wind and vertical velocity data, we examined the boundary layer characteristics associate with the sea breeze front and after the passage of the front at Lamezia Terme site during the 15 July 2009. In the present case, similar features to the other days of breeze regime are observed, as discussed below.

**Figure 11:**
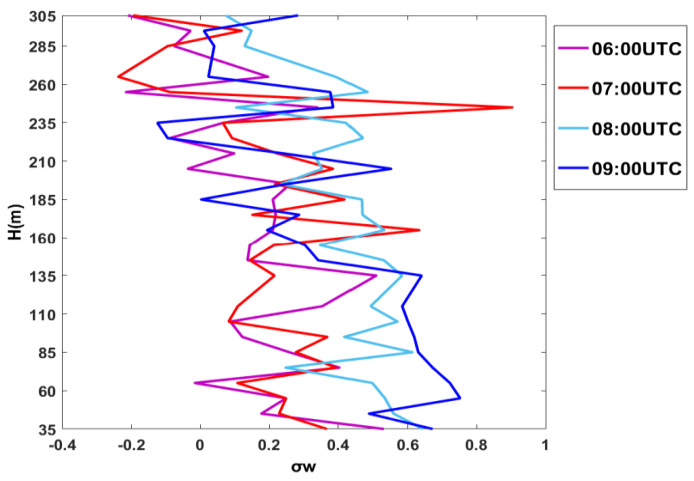
Vertical profiles of the standard deviation of the vertical wind velocity, σ_w_, during and after the sea breeze frontal passage during 15 July 2009. The sea breeze onset was at 07:00 UTC.

In Figure 11, we show the standard deviation of the vertical wind velocity, σ_w_, for 15 July 2009. A maximum around 100 m can be noted during the passage of the front.

Analyzing the vertical profiles of σ_w,_ in Figure 11, around the sea breeze front passage at 07:00 UTC, we note that both σ_w_ and the temperature at 10 m increase, (Figure 2—mast measurements), while the z/L turns to negative values indicating a change towards unstable conditions.

At the same time, the vertical profiles of σ_w_, decrease with height reaching a minimum up to 60–85 m, likely due to the low turbulence in the early stable morning; at 09:00 UTC, the convection starts to develop a layer with a constant σ_w_ up to 135–160 m where all profiles converge.

After the onset of the breeze, the advection starts to develop a stationary IBL that must interact with the convection due to the solar radiation. It is likely that the 135 m depth represents the height of the equilibrium of a convective IBL.

After the sea breeze front increased, the values of σ_w_ contributed to the amount of the kinetic energy and the growth of convective internal boundary layer with the z/L. The sodargrams in Figure 9 for the 15th of July seem to confirm this.

## 4. Conclusions

We present a unique database and a study of the development of the vertical structure of the ABL at a coastal site, located 600 m inland from the shoreline, during an experimental campaign performed in July/August 2009. We integrate surface mean and turbulence measurements with vertical wind profiles, from ground-based remote sensing devices based on different physical principles: LiDAR and SODAR. The LiDAR signal depends on the distribution of the aerosol vertical concentrations whilst the SODAR signal depends on the thermal vertical structure influencing the air density.

In the study area, both synoptic and sea breeze regimes are westerly. The sea breeze always develops, modulating the synoptic flow while the land breeze is overdriven by the synoptic flow that blows from the opposite direction.

On one hand, the integration of different instruments gave us the possibility to investigate the development of the vertical structure of the coastal area with respect to the mechanical and thermos-dynamical properties of the atmosphere, e.g., aerosols and temperature respectively; on the other hand, different meteorological situations also gave us the chance to investigate the performance of the different instruments.

The campaign was completely simulated with a NWP model.

We observed that, when synoptic conditions are favorable to sea breezes development, the colder air masses from the sea with low content of marine aerosols are advected over land in the early morning interacting with the nighttime boundary layer. After the onset of the sea breeze, an internal boundary layer develops from the coastal discontinuity and the height of the maximum backscatter from the CEILOMETER decreases, likely due to the advection of the marine aerosols above the ABL, creating a discontinuity in the aerosol concentration and size distribution. Later in the morning, when the breeze is well developed, the convection takes over and mixes marine and continental aerosols, creating a homogeneous content of aerosols filling the convective layer.

During stationary synoptic flow with wind speed typically larger than 4 ms^−1^, marine aerosols are mixed with continental aerosols and the height of the boundary layer detected by the CEILOMETER remains constant.

We also focused on the Wind LiDAR performance. During night-time, stable conditions developed under a light land breeze and the LiDAR signal could reach 250 m, often detecting a low-level jet confirmed by the SODAR measurements. During daytime, with stationary westerly synoptic winds, the LiDAR signal reached the maximum measurement height; during sea breeze days, at the onset of the breeze, the Wind LiDAR vertical wind profile rarely reached values higher than 180 m. We believe that the sea breeze advection of marine aerosols causes a nonhomogeneous columnar distribution, inducing a low LiDAR signal-to-noise ratio above the internal boundary layer. Comparisons with the CEILOMETER and SODAR measurements seem to confirm this hypothesis.

The ABL heights estimated with WRF are in good agreement with the observed ones, both for the days when synoptic flow is predominant (270°) and in breeze regime. The vertical profiles of the simulated wind speed shows values of RMSE ranging from 1.6 to 1.8 m/s, and a general overprediction (underprediction) of the wind speed, at all levels, during daytime (nighttime).

## Figures and Tables

**Table 1:** Weather conditions during the campaign.

Days in 2009	Large Scale Condition of the Weather from GFS Model	Surface Conditionfrom MAST Instrument	Weather Classification
**15–16–17 July**	A weak low-pressure field over the North Sea and a North African high-pressure field over the southern Mediterranean; winds from N-NW are driven over the Italian Peninsula	Winds intensity (WSP) are moderate (≤4 m/s)	Breeze
**18 July**	Synoptic flows become more strong and oriented from W, due to the deepening of the low-pressure on the North Sea	WSP reach 13 m/s (maximum value of the whole campaign)	Partially developed breeze
**19–20–21 July**	The low-pressure over the North Sea moves to the North Atlantic Ocean, to the west of England, and the westerly flows become less strong. The high-pressure persists on the Mediterranean, with anticyclonic reinforcement over Italy	The maximum WSP are decreasing from 7 to 4 m/s; temperatures (T) decreased slightly	Synoptic flow
**22–23 July**	The high-level flows are still from West and not exceed 6 m/s. Synoptic conditions of the previous days are persistent	The surface T are rising steadily during two days as well as the WSP	Partially developed breeze
**24–25 July**	Atlantic anticyclonic reinforcement	25th July is the day where the higher T were recorded (>33 °C)	Breeze
**26–27 July**	A slight weakening of the high-pressure over the Mediterranean Basin, with high-level flows from NW	Small differences of surface parameters are recorded compared to the previous days	Partially developed breeze
**28–29–30 July**	Similar synoptic situation of last days, with persistent winds from the N-W in the Mediterranean Basin	The trend of the WSP and surface T is constant over the 3 days, with an evident increasing westerly winds contribution, starting from the daily central hours	Breeze
**31 July–1 August**	Similar synoptic situation, with persistent winds from the N-W in the Mediterranean Basin	WSP and surface T values non exceeding 5 m/s and 29 °C respectively	Partially developed breeze
**2–3–4 August**	Similar synoptic situation. Westerly flows are still driven by the low-pressure over the North Atlantic Ocean	T are initially in the gradual growth; on the August 3 they reach the higher value (>33 °C), WSP ≥ 6 m/s	Breeze
**5–6 August**	Similar synoptic situations	Temperatures and wind conditions persist in similar situation	Partially developed breeze

**Table 2:** Comparison between wind speeds measured by LiDAR and SODAR at different heights, available data and correlations.

Heights SODAR (m)	Heights LiDAR (m)	R	Number of Data
35	40	0.84	1090
45	40	0.84	1090
75	80	0.85	910
85	80	0.84	910
115	120	0.8	768
125	120	0.78	768
155	160	0.75	533
165	160	0.73	533
215	220	0.63	153
225	220	0.59	153

**Table 3:** Estimated averages of Height of the Boundary Layer (HBL) from CEILOMETER data for synoptic and breeze conditions classified accordingly night and day hours by the GM and IBM methods and IBM method classified in stability classes.

	Synoptic		Breeze	
	HBL-Night (m)	HBL-Day (m) (7––18 UTC)	HBL-Night (m)	HBL-Day (m) (7–18 UTC)
**GM (Mean All Backscatter Profiles During Hourly Night/Day)**	680	580	180	460
**IBM (Mean All Profiles During Hourly Night/Day)**	660	570	160	420
**IBM (Classified in Stability Classes)**	640 Near-Neutral	560 Near Neutral	140 Very Stable	400 Unstable
**N° Profiles**	126	97	131	106

**Table 4:** Comparison between the mean HBL at 12:00 UTC from the IBM and GM methods and from WRF.

	Synoptic	Breeze
	HBL-Day (m) (12 (UTC))	HBL-Day (m) (12 (UTC))
**GM (Mean All Backscatter Profiles At 12 UTC) CEILOMETER Data**	590	470
**IBM (Mean All Profiles At 12 UTC) CEILOMETER Data**	810	484
**WRF (Mean All Values At 12 UTC Simulated By The Model)**	755	493

## References

[1] Emeis S., Schafer K., MÄunkel C. (2008). Surface-based remote sensing of the mixing-layer height a review. Meteorol. Z..

[2] Helmis C.G. (2007). An experimental case study of the mean and turbulent characteristics of the vertical structure of the atmospheric boundary layer over the sea. Meteorol. Z..

[3] Emeis S., Münkel C., Vogt S., Muller W.J., Schafer K. (2004). Atmospheric boundary-layers structure from simultaneous SODAR, RASS, and ceilometer measurements. Atmos. Environ..

[4] Münkel C., Eresmaa N., Räsänen J., Karppinen A. (2007). Retrieval of mixing height and dust concentration with lidar ceilometer. Bound.Layer Meteorol..

[5] Contini D., Grasso F., Mastrantonio G., Viola A.P., Martano P. (2007). Performances of a modular PC-based Multi-Tone Sodar system in measuring vertical wind velocity. Meteorol. Z..

[6] Mastrantonio G., Petenko I., Viola A., Argentini S., Coniglio L., Monti P., Leuzzi G. (2008). Influence of the synoptic circulation on the local wind field in a coastal area of the Tyrrhenian Sea. Earth Environ. Sci..

[7] Barantiev D., Batchvarova E., Novitsky M. (2017). Breeze circulation classification in the coastal zone of the town of Ahtopol based on data from ground based acoustic sounding and ultrasonic anemometer. Bulg. J. Meteorol. Hydrol..

[8] Lyulyukin V., Kallistratova M., Zaitseva D., Kuznetsov D., Artamonov A., Repina I., Petenko I.V., Kouznetsov R., Pashkin A. (2019). Sodar Observation of the ABL Structure and Waves over the Black Sea Offshore Site. Atmosphere.

[9] Peña A., Gryning S.-E., Hahmann A.N. (2013). Observations of the atmospheric boundary layer height under marine upstream flow conditions at a coastal site. J. Geophys. Res. Atmos..

[10] Dang R., Yang Y., Hu X.-M., Wang Z., Zhang S. (2019). A Review of Techniques for Diagnosing the Atmospheric Boundary Layer Height (ABLH) Using Aerosol Lidar Data. Remote. Sens..

[11] Clifton A., Clive P., Gottschall J., Schlipf D., Simley E., Simmons L., Stein D., Trabucchi D., Vasiljević N., Würth I. (2018). IEA Wind Task 32: Wind Lidar Identifying and Mitigating Barriers to the Adoption of Wind Lidar. Remote. Sens..

[12] Peña A., Floors R., Sathe A., Gryning S.-E., Wagner R., Courtney M.S., Larsén X.G., Hahmann A.N., Hasager C.B. (2015). Ten Years of Boundary-Layer and Wind-Power Meteorology at Høvsøre, Denmark. Bound. Layer Meteorol..

[13] Tsaknakis G., Papayannis A., Kokkalis P., Amiridis V., Kambezidis H.D., Mamouri R.E., Georgoussis G., Avdikos G. (2011). Inter-comparison of lidar and ceilometer retrievals for aerosol and Planetary Boundary Layer profiling over Athens, Greece. Atmospheric Meas. Tech. Discuss..

[14] Peña A., Gryning S.E., Mann J. (2010). On the length scale of the wind profile. Quart. J. Roy. Meteor. Soc..

[15] Sempreviva A.M., Gryning S.-E. (2000). Mixing height over water and its role on the correlation between temperature and humidity fuctuations in the unstable surface layer. Bound.-Lay. Meteorol..

[16] Skamarock W.C., Klemp J.B., Dudhia J., Gill D.O., Barker D.M., Duda M.G., Huang X.Y., Wang W., Powers J.G. (2008). A Description of the Advanced Research WRF Version 3. NCAR Tech. Note NCAR/TN-475+STR.

[17] Avolio E., Federico S., Miglietta M.M., Lo Feudo T., Calidonna C.R., Sempreviva A.M. (2017). Sensitivity analysis of WRF model ABL schemes in simulating boundary-layer variables in southern Italy: An experimental campaign. Atmos. Res..

[18] Avolio E., Federico S. (2018). WRF simulations for a heavy rainfall event in southern Italy: Verification and sensitivity tests. Atmospheric Res..

[19] Iacono M.J., Delamere J.S., Mlawer E.J., Shephard M.W., Clough S.A., Collins W.D. (2008). Radiative forcing by long-lived greenhouse gases: Calculations with the AER radiative transfer models. J. Geophys. Res. Space Phys..

[20] Chou M.D., Suarez M.J. (1999). A solar radiation parameterization for atmospheric studies. NASA Tech. Memo..

[21] Tewari M., Chen F., Wang W., Dudhia J., LeMone M.A., Mitchell K., Ek M., Gayno G., Wegiel J., Cuenca R.H. Implementation and verification of the unified NOAH land surface model in the WRF model. Proceedings of the 20th Conference on Weather Analysis and Forecasting.

[22] Hong S.Y., Dudhia J., Chen S.H. (2004). A revised approach to ice microphysical processes for the bulk parameterization of clouds and precipitation. Mon. Wea. Rev..

[23] Kain J.S. (2004). The Kain–Fritsch convective parameterization: An update. J. Appl. Meteor..

[24] Eresmaa N., Karppinen A., Joffre S.M., Rsnen J., Talvitie H. (2006). Mixing height determination by ceilometer. Atmos. Chem. Phys..

[25] Steyn D.G., Baldi M., Hoff R.M. (1999). The detection of mixed layer depth and entrainment zone thickness from LiDAR backscatter profiles. J. Atmos. Oceanic Technol..

[26] Peña A., Gryning S.E., Hasager C.B. (2010). Comparing mixing-length models of the diabatic wind profile over homogeneous terrain. Theor. Appl. Climatol..

[27] Shafer K., Emeis S.M., Rauch A., Mounkel C., Vogt S. (2004). Determination of mixing layer heights from ceilometer data. Remote. Sens. Clouds Atmos. IX.

[28] Federico SPasqualoni L., Sempreviva A.M., De Leo L., Avolio E., Calidonna C., Bellecci C. (2010). The seasonal characteristics of the breeze circulation at a coastal Mediterranean site in South Italy. Adv. Sci. Res..

[29] Wandinger U., Freudenthaler V., Baars H., Amodeo A., Engelmann R., Mattis I., Groß S., Pappalardo G., Giunta A., D’Amico G. (2016). EARLINET instrument intercomparison campaigns: Overview on strategy and results. Atmos. Meas. Tech..

[30] Floors R., Vincent C.L., Gryning S.E., Peña A., Batchvarova E. (2013). The wind profile in the coastal boundary layer: Wind lidar measurements and numerical modelling. Bound.-Layer Meteorol..

[31] Floors R., Peña A., Gryning S.E. (2015). The effect of baroclinicity on the wind in the planetary boundary layer. Quart. J. Roy. Meteor. Soc..

[32] Neff W.D., Coulter R.L., Lenschow D.H. (1986). Acoustic remote sensing. Probing the Atmospheric Boundary Layer.

[33] Kallistratova M.A., Petenko I.V., Kouznetsov R.D., Kulichkov S.N., Chkhetiani O.G., Chunchusov I.P., Lyulyukin V.S., Zaitseva D.V., Vazaeva N.V., Kuznetsov D.D. (2018). Sodar Sounding of the Atmospheric Boundary Layer: Review of Studies at the Obukhov Institute of Atmospheric Physics, Russian Academy of Sciences. Izv. Atmos. Ocean. Phys..

